# Obstructive sleep apnea and oral language disorders^☆^

**DOI:** 10.1016/j.bjorl.2016.01.017

**Published:** 2016-04-28

**Authors:** Camila de Castro Corrêa, Maria Gabriela Cavalheiro, Luciana Paula Maximino, Silke Anna Theresa Weber

**Affiliations:** a Universidade Estadual Paulista “Júlio de Mesquita Filho” (FM–UNESP), Faculdade de Medicina de Botucatu, Departamento de Oftalmologia e Otorrinolaringologia, Botucatu, SP, Brazil; b Universidade de São Paulo (FOB–USP), Faculdade de Odontologia de Bauru, Departamento de Fonoaudiologia, Bauru, SP, Brazil

**Keywords:** Child language, Language disorders, Speech, language and hearing sciences, Obstructive sleep apnea, Linguagem infantil, Transtornos da linguagem, Fonoaudiologia, Apneia do sono tipo obstrutiva

## Abstract

**Introduction:**

Children and adolescents with obstructive sleep apnea (OSA) may have consequences, such as daytime sleepiness and learning, memory, and attention disorders, that may interfere in oral language.

**Objective:**

To verify, based on the literature, whether OSA in children was correlated to oral language disorders.

**Methods:**

A literature review was carried out in the Lilacs, PubMed, Scopus, and Web of Science databases using the descriptors “Child Language” AND “Obstructive Sleep Apnea”. Articles that did not discuss the topic and included children with other comorbidities rather than OSA were excluded.

**Results:**

In total, no articles were found at Lilacs, 37 at PubMed, 47 at Scopus, and 38 at Web of Science databases. Based on the inclusion and exclusion criteria, six studies were selected, all published from 2004 to 2014. Four articles demonstrated an association between primary snoring/OSA and receptive language and four articles showed an association with expressive language. It is noteworthy that the articles used different tools and considered different levels of language.

**Conclusion:**

The late diagnosis and treatment of obstructive sleep apnea is associated with a delay in verbal skill acquisition. The professionals who work with children should be alert, as most of the phonetic sounds are acquired during ages 3–7 years, which is also the peak age for hypertrophy of the tonsils and childhood OSA.

## Resumo

**Introdução:**

Crianças e adolescentes com Apneia Obstrutiva do Sono (AOS) podem apresentar sonolência diurna, alterações de aprendizado, memória e atenção, que podem interferir na linguagem oral.

**Objetivo:**

Verificar, com base na literatura, se a AOS apresenta correlação com alterações da linguagem oral.

**Método:**

Foi realizada revisão bibliográfica nas bases de dados Lilacs, Pubmed, Scopus e Web of Science, a partir das palavras-chaves “Linguagem Infantil” AND “Apneia do Sono Tipo Obstrutiva”. Os artigos que não se relacionavam ao tema foram excluídos, bem como estudos com crianças que apresentassem outras comorbidades, além da AOS.

**Resultados:**

Foram localizados 0 artigos na base Lilacs, 37 na Pubmed, 47 na Scopus e 38 na Web of Science. A partir dos critérios de inclusão e exclusão, foram selecionados seis estudos, publicados em 2004 a 2014. Dos artigos incluídos, observou-se em quatro artigos a relação do grupo com ronco primário/SAOS com a Linguagem Receptiva e em quatro artigos a relação dessa população com a Linguagem Expressiva. Ressalta-se que os artigos utilizaram instrumentos diferentes e consideraram níveis diversificados da Linguagem.

**Conclusão:**

O diagnóstico e tratamento tardio de AOS resultam em alterações significantes na qualidade da aquisição verbal. Torna-se imprescindível a atenção dos profissionais que atuam com o a população infantil para este aspecto, uma vez que grande parte dos sons da fala são adquiridos entre 3–7 anos, que corresponde ao período de pico de ocorrência de hipertrofia adenoamigdaliana e AOS na infância.

## Introduction

Obstructive Sleep Apnea (OSA) is characterized by partial and/or complete upper airway obstruction during sleep, associated with increased respiratory effort, fragmented sleep, and/or gas exchange abnormalities.^1^, ^2^ There are differences in what is observed in adults *versus* children with respect to pathophysiology, clinical features and treatment.^2^ The pathophysiology of OSA in children is associated with a predominant pattern of partial and persistent upper airway obstruction, resulting in hypercapnia and intermittent hypoxia.^3^ Snoring, the main symptom of OSA, is present in the clinical picture of almost all children with the alteration. Other signs and symptoms such as forced mouth breathing with costal retractions, sleepwalking, enuresis and night sweats, coughing, gagging, and agitation during sleep are also part of the clinical picture, and it is common for these children to move around in search of positions that facilitate the passage of air.^4^ Treatment differs from that of adults: adenotonsillectomy is considered the gold standard treatment and, when performed for the proper indications, it benefits the child with respect to neuropsychological, behavioral, and quality of life issues; obese children exhibit a lower rate of success.^5^, ^6^

It is estimated that the prevalence of OSA in healthy children without other associated clinical picture varies from 0.7% to 3%.^7^, ^8^, ^9^, ^10^ The incidence is higher in the preschool range, an age when there is a greater disproportion between the hypertrophy of the palatine and pharyngeal tonsils and upper airway dimensions.^5^ This period is also recognized as privileged for the acquisition and development of language and intense neuroplasticity of the central nervous system, which favors learning.^11^, ^12^, ^13^, ^14^

Among the consequences of OSA in children, the association with attention and memory deficits must be considered; that could impair information processing and recording, decreasing the learning capacity.^15^, ^16^, ^17^ The condition also affects the mood, expressive language skills, school performance, cognitive skills, and visual perception of this population.^18^, ^19^, ^20^

Because the reported frequency of OSA in the literature occurs during an important phase of development in preschool children and OSA's effect on skills involved in the language acquisition process, learning, and school performance, it is relevant to assess the development of oral language in these children. There is strong evidence of OSA association with neurocognitive deficits,^6^, ^17^, ^19^ but studies that specifically focused on the development of language were not retrieved from the literature.

To understand oral language in this population, psycholinguistic skills must be investigated broadly, from the receptive language, which is defined as the capacity to understand the language in different aspects, such as understanding the tone of each other's voice during speech and the meaning of the words, when these are used in different contexts and complexities; to the expressive language, which refers to the capacity of organizing the linguistic system, in motor programming; and finally, in the verbalization of a sequence of symbols and meanings, in the case of oral language, resulting in the capacity to express oneself verbally.^21^, ^22^, ^23^

The observation and measurement of all these linguistic levels can only be achieved through the application of protocols specifically developed for the patient's native language that have comparative scores with normative data for each age group. The only study detailing this aspect is a systematic review of the following tests used to assess receptive oral language: the Peabody Picture Vocabulary Test, Peabody Picture Vocabulary Test-Revised (PPVT-R), Swedish Communication Screening at 18 months of act (SCS18), Test for Reception of Grammar-2 (TROG-2), Reynell Test, Reynell Language Development Scales, and Reynell Developmental Language Scales-II. It also emphasized that there are few tools and not all of them have validity studies.^24^

Therefore, this study aimed to verify whether the presence of OSA is associated with possible oral language alterations.

## Methods

A literature search was carried out with no temporal limitation, using the keywords “Child Language” AND “Obstructive Sleep Apnea”, as well as their counterparts in Portuguese, “Linguagem Infantil” AND “Apneia do Sono Tipo Obstrutiva”. The search was performed in four databases: Lilacs, PubMed, Scopus, and Web of Science.

The inclusion criteria comprised articles written on the central topic of children/adolescents with OSA, with focus on oral language alterations. Thus, the exclusion criteria included: articles that assessed other concomitant medical conditions that justified sleep or language alterations, such as cleft lip and palate, genetic syndromes (Down, craniosynostosis, and velocardiofacial syndrome), and ADHD; those with focus on motor speech disorders, such as speech apraxia; and literature review articles. It is noteworthy that the search was carried out using the VPN (Virtual Private Network) system and articles that were not fully available were also excluded.

Article selection was carried out by reading the titles and abstracts. Subsequently, the articles were analyzed in full, after which they were definitively included or not in the review. The articles included in the review were analyzed regarding their objectives, methods, results, and conclusions. The specific results of the evaluations regarding oral language, evaluated oral language specification (receptive and/or expressive) were also analyzed, and the limitations of each study were identified.

## Results

The search found no articles in Lilacs, 37 in PubMed, 47 in Scopus, and 38 in Web of Science databases.

**Figure 1:**
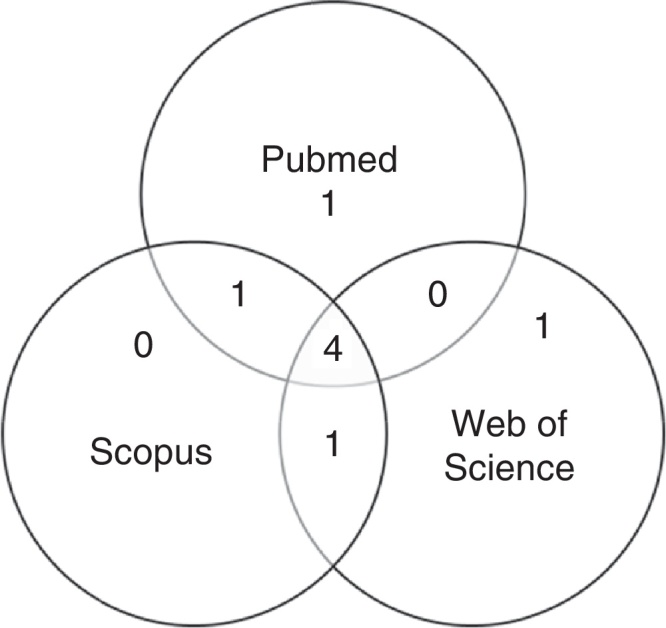
Database description of the abstracts considered for the review, in numbers, also showing when they were found in more than one database.

After first analysis, reading the titles and abstracts, eight studies were selected. The location in one or more databases where the articles were found is shown in Fig. 1.

For the final inclusion, all articles were read in full, except two, whose full versions were not available and thus were excluded. Therefore, Table 1 shows the six studies included in this study, with information on authorship, year, journal, and database from where they were retrieved, shown in ascending chronological order.

**Table 1:** Data on authorship, year, journal, and database of assessed articles.

Authors	Year	Journal	Database
O’Brien et al.^25^	2004	Pediatrics	PubMed – Web of science – Scopus
Kurnatowski et al.^26^	2006	Int J Pediatr Otorhinolaryngol	PubMed – Web of science – Scopus
Andreou and Agapitou^27^	2007	Archives of Clinical Neuropsychology	Web of science
Landau et al.^28^	2012	Pediatric Pulmonology	PubMed
Liukkonen et al.^29^	2012	Int J Pediatr Otorhinolaryngol	PubMed – Web of science – Scopus
Yorbik et al.^30^	2014	Sleep and Biological Rhythms	Web of science – Scopus

Table 2 shows the analysis of the included articles.

**Table 2:** Information on the objective, sample, methods, and results (specifically regarding oral language) of the analyzed articles.

Author, year	Objective	Sample	Methods – focus on oral language	OSA diagnostic criteria	Results – focus on oral language	Receptive and/or expressive language	Study limitation
Study design							
O’Brien et al., 2004^25^	To evaluate the association of primary snoring and neurobehavioral deficits in children.	87 children with primary snoring and 31 healthy subjects, aged 5–7 years.	Used the NEPSY.	Diagnosis of Primary Snoring by PSG, considering the AI < 1; AHI < 5 and no abnormal alterations in gas exchange.	Language showed significantly lower results for the primary snoring group when compared to the control group.	Receptive and expressive language	It did not perform tests to assess hearing.
Cross-sectional							
Kurnatowski et al., 2006^26^	To analyze neurocognitive disorders (sensory-motor coordination, perception, memory, learning, concentration, focused attention and language reception) in children with OSA due to adenotonsillar hypertrophy.	221 children in total. 117 children with OSAS: 87 aged 6–9 years and 34 aged 10–13 years. 104 healthy children.	Token Test (TT) – to assess the level of sensorimotor integration, perception and receptive language processes.	Diagnosis of OSAS by PSG with AHI > 1, oxygen desaturation < 92%.	The groups of children with OSAS had results below those found in healthy children regarding Receptive Language.	Receptive Language	It did not perform tests to assess hearing.
Cross-sectional							
Andreou and Agapitou, 2007^27^	To analyze whether OSA in childhood may be related to verbal fluency and academic performance.	40 adolescents: 20 with OSA and 20 from the control group. Mean age: 18.41 years.	Two standardized tests of verbal fluency in Greek, regarding the semantic and phonological aspects.	OSA diagnosis by PSG, with AHI > 10 and/or SaO_2_ < 95% per event, and heart rate > 60 beats per minute.	A difference was observed in the phonological and semantic aspects when comparing children with and without OSA. The adolescents with OSA showed worse results.	Expressive Language	It did not perform tests to assess hearing and cognition.
Cross-sectional							
Landau et al., 2012^28^	To analyze the hypothesis that behavioral and cognitive functions of preschool children with OSA are impaired when compared to healthy children. To verify whether there was improvement after adenotonsillectomy.	45 children with OSA and 26 healthy children aged 2.5–5 years.	The test Kaufman Assessment Battery for Children (K-ABC) was applied.	Diagnosis of OSA by PSG com AHI > 1.	Before surgery, the group with OSA showed worse performance in verbal fluency, and after surgery, there was an improvement in this regard.	Expressive language	It did not perform tests to assess hearing.
Cross-sectional							
Liukkonen et al., 2012^29^	To assess the association between sleep-disordered breathing and cognitive function in children.	44 children with primary snoring and 51 healthy ones, aged 1–6 years.	The NEPSY assessment tool (comprehension of instructions, speeded naming and body part naming).	Diagnosis of Primary snoring by PSG, with AHI < 1. Hypopnea was defined as an airflow volume reduction of <50%, followed by awakening, oxyhemoglobin desaturation >2%.	The group of children with primary snoring obtained the lowest scores in language functions (comprehension of instructions, speeded naming).	Receptive and expressive language	It did not perform tests to assess hearing.
Cross-sectional							
Yorbik et al., 2014^30^	To investigate the effects of snoring and fragmented sleep on mental development in preschool children.	212 children, 37 with complaints of snoring and 25 with fragmented sleep complaints, aged 3.1–6 years.	Peabody Picture Vocabulary Test was used.	Through a questionnaire.	Children with complaints of snoring and with fragmented sleep had lower scores on language.	Receptive Language	It did not perform PSG assessment and did not assess hearing.
Cross-sectional							

PSG, polysomnography; AHI, apnea-hypopnea index; AI, apnea index.

## Discussion

A key feature of current studies on OSA is an interdisciplinary approach reflecting the varied and heterogeneous impairments that this condition may cause; treatment requires a holistic view of the individual for greatest effectiveness.

During this search, we observed that the selected articles on ORAL LANGUAGE were published only recently. The diagnosis of OSA has increased in recent years,^31^ which may explain the increase in the number of children with OSA and the higher number of current scientific research investigating these aspects.

Most studies were published in pediatric journals (four), one in sleep medicine, and one in neuropsychology. It is noteworthy that there were no publications in speech therapy and audiology journals, *i.e.*, those professional responsible for the understanding and speech therapy aspects of the peripheral and central auditory function, vestibular function, oral and written language, voice, fluency, speech articulation and myofunctional, orofacial, cervical, and deglutition systems.^32^

In general, the assessed studies evaluated behavioral and neurocognitive functions; one study analyzed verbal fluency and academic performance. Thus, there were no studies that exclusively analyzed oral language, but rather tried to effectively understand language at all levels. For the understanding of oral language, the abilities of Expressive and Receptive Language should be considered, that is, the thought organization and expression processes that, as well-organized behavior, can be described by the aspects: phonological (inventory of sounds of a language and the combination of rules to form meaningful units); syntactic (verbal production rules as a structure, taking into account the morphological and grammatical analysis); semantic (characterized by the lexical repertoire and related to the meaning of words and their combinations); and pragmatic (rules related to intentionality, context, and function of speech).^33^, ^34^, ^35^, ^36^

Moreover, considering that the development of language occurs gradually, respecting the child's maturation process and influenced by the associations established with the environment where the child lives,^32^ the high variability of the age range of the subjects included in the studies analyzed in this review was a limiting factor, this prevented comparisons among the studies. Three studies assessed children younger than 6 years,^27^, ^29^, ^30^ one assessed children aged 5–7 years,^25^ another assessed children aged 6–13 years,^26^ and one study assessed adolescents.^28^

The development of language is characterized by the presence of some markers, one of which is age from 4 to 7 years, when the child gradually starts to produce more complex sounds, starting with the appropriate production of simpler words progressing to longer words.^35^ Regarding the samples assessed in the studies, the maximum age of 7 years was observed in four of them, and the other two considered children that were older than the expected age for the stability of the phonological system. Although it is not possible to establish associations between the samples regarding the phonological development due to the age range, it should be noted that the period between 3 and 7 years is the peak of adenoid hypertrophy in children with OSA,^37^ and it is also when most speech sounds are acquired.^35^

The studies also differ regarding the sleep characteristics, as three of them analyzed children with OSA assessed by polysomnography (PSG), two analyzed children with primary snoring, and one study did not include PSG among their assessment methods, characterizing the sample only through questionnaires. The definition of OSA diagnosis by PSG and its degree, is necessary to allow for the correlation of changes in oral language with the evaluation of physiological impairment.^38^ Moreover, of the five studies that included PSG among their assessment methods, the criteria/parameters utilized to consider OSA were also different (with AHI ranging from >1 to >10). Thus, it is difficult to compare the included studies and considering that all of them had a cross-sectional design, their level of evidence is an intermediate one.

Regarding the methodology of language analysis through the different tests used to assess oral language (Kaufman, Peabody, Token, NEPSY, and an unspecified Greek test), it was not possible to perform a more thorough comparison of the outcomes, suggesting the need for studies with the standardization of these protocols, to provide a better understanding of the correlation between OSA and oral language. However, despite the absence of statistical indices comparing the results of the present investigation, there is growing evidence of oral language impairment in OSA cases.

Among the oral language levels, the results of the aforementioned studies showed difficulties in the semantic, phonological, and verbal fluency levels. Some authors have tried to explain how the neurocognitive performance of children may be affected by sleep alterations. Furthermore, it has been stated that language deficits and verbal fluency can be explained by the cumulative effect of sleep architecture disruption associated with the neurological maturation period, which over a period of a few years interferes with the development of neuronal synaptic networks, occurring rapidly and intensively in children.^19^, ^39^ Verbal fluency deficits are also associated with prefrontal cortex dysfunction.^40^, ^41^

Therefore, the early diagnosis and treatment of OSA should be emphasized, not only because of the possible implications for oral language, as demonstrated in the reviewed studies, which tend to worsen as the chronological age increases,^27^ but also for the benefits in neurocognitive performance and quality of life of these children.^18^, ^42^, ^43^, ^44^

## Conflicts of interest

The authors declare no conflicts of interest.
